# The prognostic value of lymph node ratio in comparison to positive lymph node count in penile squamous cell carcinoma

**DOI:** 10.1007/s11255-021-02996-3

**Published:** 2021-09-28

**Authors:** Jiajie Yu, Qian Long, Zhiqiang Zhang, Shufen Liao, Fufu Zheng

**Affiliations:** 1 Department of Urology, The First Affiliated Hospital, Sun Yat-Sen University, No.58 the 2nd Zhongshan Road, Guangzhou, 510080 China; 2 State Key Laboratory of Oncology in South China; Collaborative Innovation Center of Cancer Medicine, Sun Yat-Sen University Cancer Center, Guangzhou, China

**Keywords:** Lymph node ratio, Positive lymph node count, Penile squamous cell carcinoma, SEER

## Abstract

**Purpose:**

Penile cancer is a rare male neoplasm with a wide variation in its global incidence. In this study, the prognostic value of lymph node ratio (LNR) was compared to that of positive lymph node count (PLNC) in penile squamous cell carcinoma.

**Methods:**

A total of 249 patients with penile squamous cell carcinoma were enrolled from The Surveillance, Epidemiology, and End Results (SEER) database between 2010 and 2015. The X-tile program was used to calculate the optimal cut-off values of LNR and PLNC that discriminate survival. We used the χ^2^ or the Fisher exact probability test to assess the association between clinical-pathological characteristics and LNR or PLNC. Univariate and multivariate Cox regression analyses were performed to identify independent prognostic factors for survival. Spearman correlation analysis was used to determine the correlation between LNR and PLNC.

**Results:**

We found that patients with high LNR tended to have advanced N stage, the 7th AJCC stage, and higher pathological grade, while patients with high PLNC had advanced N stage and the 7th AJCC stage. Univariate Cox regression analysis revealed that the N stage, M stage, the 7th AJCC stage, lymph-vascular invasion, LNR, and PLNC were significantly associated with prognosis. Multivariate Cox regression analysis demonstrated that LNR rather than PLNC was an independent prognostic factor for cancer-specific survival. Subgroup analysis of node-positive patients showed that LNR was associated with CSS, while PLNC was not.

**Conclusion:**

LNR was a better predictor for long-term prognosis than PLNC in patients with penile squamous cell carcinoma.

## Introduction

Penile cancer (PC) is a relatively rare disease in developed countries, with approximately 2200 new cases of PC reported in 2020 in the US [1]. However, PC remains a significant public health concern since it has a considerably higher incidence in developing countries [2, 3]. Besides, PC is most common in men aged between 50 and 70 [4]. Pathologically, squamous cell carcinoma (SCC) is the most common type of penile cancer, accounting for approximately 95% of malignant neoplasms of the penis, although other histological types have also been reported [5]. Patients with early-stage PC generally have a favorable prognosis; however, the 5-year cancer-specific survival precipitously declines with lymph node metastasis [6]. Therefore, more effective therapeutic strategies and better prognostic predictors are needed for PSCC patients.

The TNM staging system is one of the most important prognostic factors for survival, associated with tumor (T)/lymph node metastasis (N)/distant metastasis (M) in cancers. Recently, the lymph node ratio (LNR, the ratio of metastatic to total examined lymph nodes) and positive lymph node count (PLNC, the number of metastatic lymph nodes) have been considered as powerful prognostic factors in various tumors [7–12]. However, the prognostic value of LNR versus PLNC in penile squamous cell carcinoma (PSCC) has not been well established.

Therefore, this study aimed to compare the prognostic impact of PLNC versus LNR in PSCC patients.

## Methods

### Patients and variables

**Fig. 1:**
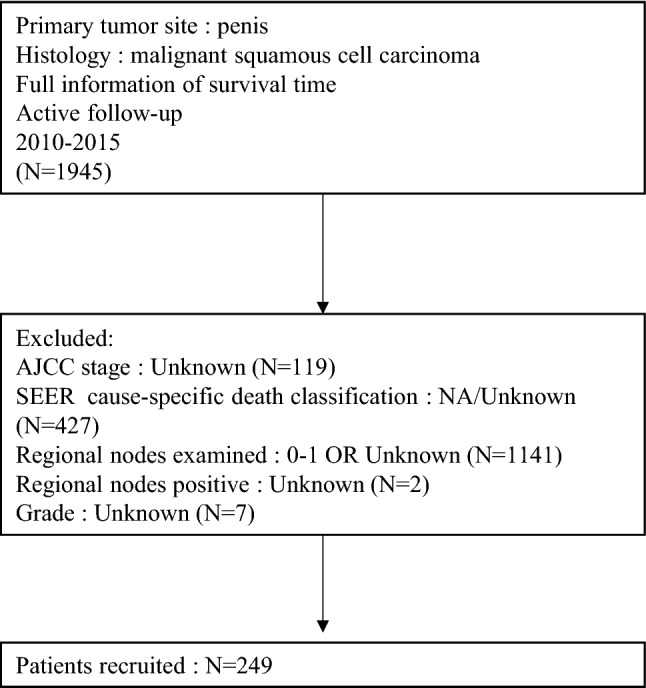
Flow diagram indicating patients from the SEER database

A total of 249 men diagnosed with PSCC between 2010 and 2015 were retrospectively identified using the SEER*Stat software program. The inclusion criteria was as follows: (1) ICD-O-3 topography code of primary tumor site: C60.0, C60.1, C60.2, C60.8, C60.9; (2) ICD-O-3 histology code of malignant squamous cell carcinoma: 8051, 8052, 8070–8076, 8081, 8083 and 8084; (3) Complete survival time information; (4) Active follow-up; (5) Diagnostic period: 2010–2015. The exclusion criteria were as follows: (1) AJCC stage: Unknown; (2) SEER cause-specific death classification: NA/Unknown; (3) Regional nodes examined: 0–1 OR Unknown; (4) Regional nodes positive: Unknown; (5) Grade: Unknown. The screening process is as presented in Fig. 1.

The following variables were assessed: age, marital status, the 7th AJCC/TNM stages, histology, grade, primary site, lymph-vascular invasion, PLNC, and LNR. The age was grouped by patients’ median age at diagnosis. Detailed information is as shown in Table 1. The endpoints of this study were overall survival (OS) and cancer-specific survival (CSS), which were determined by vital status and SEER cause-specific death classification, respectively.

**Table 1:** Characteristics of patients recruited from SEER

Characteristic	Patients, No.(%)(*N* = 249)
Age, y	
Median (SD)	62(12.3)
≤ 62	126(50.6)
> 62	123(49.4)
Marital status	
Married	149(59.8)
Single	52(20.9)
Unknown	10(4.0)
Divorced/Separated/Widowed	38(15.3)
T stage	
T1a, T1b, T1NOS	54(21.6)
T2	107(43.0)
T3	83(33.3)
T4	5(2.0)
N stage	
N0	116(46.6)
N1	39(15.7)
N2	37(14.9)
N3	57(22.9)
M stage	
M0	240(96.4)
M1	9(3.6)
The 7th AJCC stage	
I	11(4.4)
II	101(40.6)
IIIA	36(14.5)
IIIB	35(14.1)
IV	66(26.5)
Histology	
Verrucous carcinoma	6(2.4)
Papillary squamous cell carcinoma	2(0.8)
Squamous cell carcinoma, NOS	150(60.2)
Squamous cell carcinoma, keratinizing	69(27.7)
Squamous cell carcinoma, large cell, nonkeratinizing	6(2.4)
Squamous cell carcinoma, spindle cell	5(2.0)
Basaloid squamous cell carcinoma	11(4.4)
Grade	
I-II	175(70.3)
III-IV	74(29.7)
Primary site	
Prepuce	18(7.2)
Glans penis	101(40.6)
Body of penis	16(6.4)
Overlapping lesion of penis	14(5.6)
Penis, NOS	100(40.2)
Lymph-vascular invasion	
Unknown	35(14.1)
Negative	134(53.8)
Positive	80(32.1)
LNR	
≤ 0.23	215(86.3)
> 0.23	34(13.7)
PLNC(grouped by 1)	
≤ 1	162(65.1)
> 1	87(34.9)
PLNC(grouped by 3)	
≤ 3	214(85.9)
> 3	35(14.1)

*LNR* lymph node ratio, *PLNC* positive lymph node count

### Statistical analysis

The lymph node ratio was calculated as the ratio of the number of positive lymph nodes to the total number of lymph nodes examined. The optimal cut-off value was determined using the X-tile program. For OS, the optimal cut-off value of LNR was 0.23, with values ≤ 0.23 considered low and values > 0.23 considered high. The optimal cut-off value of PLNC was 3, with values ≤ 3 regarded as low while those > 3 were high. For CSS, the optimal cut-off value of LNR was identical to OS. The optimal cut-off value of PLNC was 1, with PLNC ≤ 1 considered low, and PLNC > 1 high. The χ2 test or the Fisher exact probability test was used to assess the association between the clinical-pathological characteristics and LNR or PLNC. Kaplan–Meier method was used to determine the survival analysis and the Log-rank test was used to examine the statistical differences between LNR or PLNC groups in terms of overall survival and cancer-specific survival. Spearman correlation analysis was used to determine the correlation between LNR and PLNC. Cox regression analysis was used to compute the hazard ratios (HRs) and 95% confidence intervals (95%CIs) for the identification of the prognostic factors in the survival of PSCC patients. All statistical analyses were performed using IBM SPSS Statistics 25. *P* values < 0.05 were considered statistically significant in the χ^2^ test, the Fisher exact probability test, the Log-rank test, and multivariate Cox regression analysis, while P values < 0.1 were considered significant in univariate Cox regression analysis.

## Results

### Patients’ characteristics

A total of 249 patients with PSCC were recruited from the SEER database. Of these, 132 (53%) patients were confirmed to have lymph node metastasis, whereas 117 (47%) patients were free of lymph node metastasis. The median follow-up time was 30 months (range of 0–82 months). At the end of the follow-up, 57(22.9%) patients died from PSCC. The median number of LNR, PLNC, and lymph nodes examined was 0.04 (range of 0.00–1.00), 1(range of 1–18), and 17 (range of 2–78), respectively. A detailed description of the clinical-pathological characteristics of the enrolled patients is shown in Table 1.

### The relationship between LNR/PLNC and clinical pathological characteristics in patients with PSCC

We used the χ^2^ test or the Fisher exact probability test to compare the characteristics between the LNR/PLNC groups and clinical-pathological characteristics of PSCC patients. Our results revealed that high LNR patients tended to have advanced N stage (*P < *0.001), the 7th AJCC stage (*P < *0.001), and higher pathological grade (*P = *0.048) while no significant association was found with other characteristics. PLNC was significantly associated with the N stage (*P < *0.001) and the 7th AJCC stage (*P < *0.001) based on the cut-off value of 1 and 3, while a significant association was observed between the M stage and PLNC grouping based on the cut-off value of 1 (*P = *0.010) (Table 2). These findings suggested that both high LNR and PLNC were associated with poor clinical-pathological characteristics in PSCC. Consequently, LNR and PLNC can serve as potential prognostic factors guiding clinical decisions.

**Table 2:** The relationship between LNR/PLNC and clinical pathological characteristics in PSCC

Characteristic	LNR		*χ*^2^	*P* value	PLNC		*χ*^2^	*P* value	PLNC		*χ*^2^	*P* value
	≤ 0.23	> 0.23			≤ 1	> 1			≤ 3	> 3		
Age												
≤ 62	113 (89.7%)	13 (10.3%)	2.409	0.121	81 (64.3%)	45 (35.7%)	0.067	0.795	106 (84.1%)	20 (15.9%)	0.697	0.404
> 62	102 (82.9%)	21 (17.1%)			81 (65.9%)	42 (34.1%)			108 (87.8%)	15 (12.2%)		
Marital status												
Married	125 (83.9%)	24 (16.1%)		0.498	96 (64.4%)	53 (35.6%)		0.299	126 (84.6%)	23 (15.4%)		0.918
Single	47 (90.4%)	5 (9.6%)			35 (67.3%)	17 (32.7%)			45 (86.5%)	7 (13.5%)		
Unknown	10 (100.0%)	0 (0.0%)			9 (90.0%)	1 (10.0%)			9 (90.0%)	1 (10.0%)		
Divorced/Separated/Widowed	33 (86.8%)	5 (13.2%)			22 (57.9%)	16 (42.1%)			34 (89.5%)	4 (10.5%)		
T stage												
T1a, T1b, T1NOS	49 (90.7%)	5 (9.3%)		0.330	38 (70.4%)	16 (29.6%)		0.263	51 (94.4%)	3 (5.6%)		0.103
T2	94 (87.9%)	13 (12.1%)			73 (68.2%)	34 (31.8%)			92 (86.0%)	15 (14.0%)		
T3	67 (80.7%)	16 (19.3%)			47 (56.6%)	36 (43.4%)			67 (80.7%)	16 (19.3%)		
T4	5 (100.0%)	0 (0.0%)			4 (80.0%)	1 (20.0%)			4 (80.0%)	1 (20.0%)		
N stage												
N0	116 (100.0%)	0 (0.0%)	42.644	< 0.001*	116 (100.0%)	0 (0.0%)	179.292	< 0.001*	116 (100.0%)	0 (0.0%)	62.252	< 0.001*
N1	33 (84.6%)	6 (15.4%)			33 (84.6%)	6 (15.4%)			38 (97.4%)	1 (2.6%)		
N2	29 (78.4%)	8 (21.6%)			2 (5.4%)	35 (94.6%)			22 (59.5%)	15 (40.5%)		
N3	37 (64.9%)	20 (35.1%)			11 (19.3%)	46 (80.7%)			38 (66.7%)	19 (33.3%)		
M stage												
M0	209 (87.1%)	31 (12.9%)		0.110	160 (66.7%)	80 (33.3%)		0.010*	206 (85.8%)	34 (14.2%)		1
M1	6 (66.7%)	3 (33.3%)			2 (22.2%)	7 (77.8%)			8 (88.9%)	1 (11.1%)		
The 7th AJCC stage												
I	11 (100.0%)	0 (0.0%)		< 0.001*	11 (100.0%)	0 (0.0%)		< 0.001*	11 (100.0%)	0 (0.0%)		< 0.001*
II	101 (100.0%)	0 (0.0%)			101 (100.0%)	0 (0.0%)			101 (100.0%)	0 (0.0%)		
IIIA	31 (86.1%)	5 (13.9%)			31 (86.1%)	5 (13.9%)			35 (97.2%)	1 (2.8%		
IIIB	27 (77.1%)	8 (22.9%)			2 (5.7%)	33 (94.3%)			20 (57.1%)	15 (42.9%)		
IV	45 (68.2%)	21 (31.8%)			17 (25.8%)	49 (74.2%)			47 (71.2%)	19 (28.8%)		
Histology												
Verrucous carcinoma	6 (100.0%)	0 (0.0%)		0.330	6 (100.0%)	0 (0.0%)		0.078	6 (100.0%)	0 (0.0%)		0.332
Papillary squamous cell carcinoma	1 (50.0%)	1 (50.0%)			1 (50.0%)	1 (50.0%)			1 (50.0%)	1 (50.0%)		
Squamous cell carcinoma, NOS	131 (87.3%)	19 (12.7%)			93 (62.0%)	57 (38.0%)			130 (86.7%)	20 (13.3%)		
Squamous cell carcinoma, keratinizing	57 (82.6%)	12 (17.4%)			47 (68.1%)	22 (31.9%)			56 (81.2%)	13 (18.8%)		
Squamous cell carcinoma, large cell, nonkeratinizing,	5 (83.3%)	1 (16.7%)			2 (33.3%)	4 (66.7%)			5 (83.3%)	1 (16.7%)		
Squamous cell carcinoma, spindle cell	4 (80.0%)	1 (20.0%)			3 (60.0%)	2 (40.0%)			5 (100.0%)	0 (0.0%)		
Basaloid squamous cell carcinoma	11 (100.0%)	0 (0.0%)			10 (90.9%)	1 (9.1%)			11 (100.0%)	0 (0.0%)		
Grade												
I-II	156 (89.1%)	19 (10.9%)	3.909	0.048*	120 (68.6%)	55 (31.4%)	3.194	0.074	149 (85.1%)	26 (14.9%)	0.313	0.576
III-IV	59 (79.7%)	15 (20.3%)			42 (56.8%)	32 (43.2%)			65 (87.8%)	9 (12.2%)		
Primary site												
Prepuce	16 (88.9%)	2 (11.1%)		0.606	10 (55.6%)	8 (44.4%)		0.635	16 (88.9%)	2 (11.1%)		0.451
Glans penis	83 (82.2%)	18 (17.8%)			63 (62.4%)	38 (37.6%)			83 (82.2%)	18 (17.8%)		
Body of penis	14 (87.5%)	2 (12.5%)			10 (62.5%)	6 (37.5%)			15 (93.8%)	1 (6.3%)		
Overlapping lesion of penis	12 (85.7%)	2 (14.3%)			11 (78.6%)	3 (21.4%)			14 (100.0%)	0 (0.0%)		
Penis, NOS	90 (90.0%)	10 (10.0%)			68 (68.0%)	32 (32.0%)			86 (86.0%)	14 (14.0%)		
Lymph-vascular invasion												
Unknown	28 (80.0%)	7 (20.0%)		0.123	24 (68.6%)	11 (31.4%)	5.278	0.071	32 (91.4%)	3 (8.6%)		0.462
Negative	121 (90.3%)	13 (9.7%)			94 (70.1%)	40 (29.9%)			116 (86.6%)	18 (13.4%)		
Positive	66 (82.5%)	14 (17.5%)			44 (55.0%)	36 (45.0%)			66 (82.5%)	14 (17.5%)		

*LNR* lymph node ratio, *PLNC* positive lymph node count

*Two-sided *P* value < 0.05

### The prognostic value of LNR and PLNC for survival in patients with PSCC

**Fig. 2:**
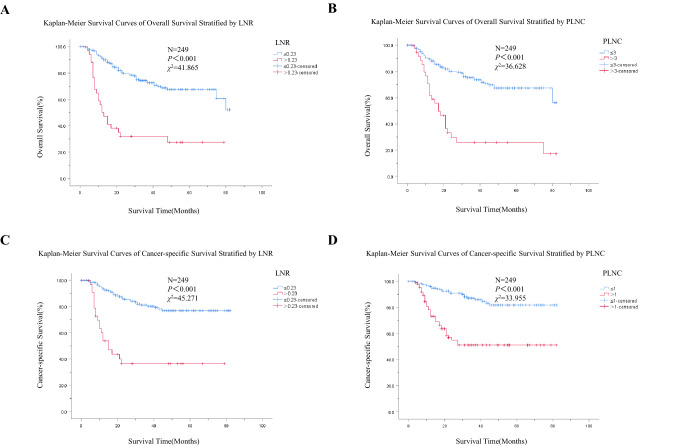
Kaplan–Meier survival curves of overall survival/cancer-specific survival **A** Overall survival analysis stratified by LNR; **B** Overall survival analysis stratified by PLNC; **C** Cancer-specific survival analysis stratified by LNR; **D** Cancer-specific survival analysis stratified by PLNC

To further explore the role of LNR and PLNC in predicting the survival of PSCC patients, Kaplan–Meier analysis and the Log-rank test were used to estimate the overall survival and cancer-specific survival based on the LNR and PLNC status. There were significant differences in overall survival analysis between the LNR (Fig. 2A) and PLNC groups (Fig. 2B). Patients with LNR ≤ 0.23 had a significantly higher 5-year overall survival rate than those with LNR > 0.23 (67.5 vs. 27.3%). The 5-year overall survival rate of patients with PLNC ≤ 3 and > 3 were 67.4 and 25.8%, respectively. A similar trend was observed in cancer-specific survival analysis (Fig. 2C and Fig. 2D). The 5-year cancer-specific survival rate of patients with LNR ≤ 0.23 was higher than LNR > 0.23 (76.9 vs. 36.4%), whereas the 5-year cancer-specific survival rate of patients with PLNC ≤ 1 and > 1 were 81.7 and 51.1%, respectively. Even for the *P* values < 0.001, the χ^2^ values of LNR were higher than PLNC, indicating that LNR may be a more promising prognostic factor for PSCC patients.

**Fig. 3:**
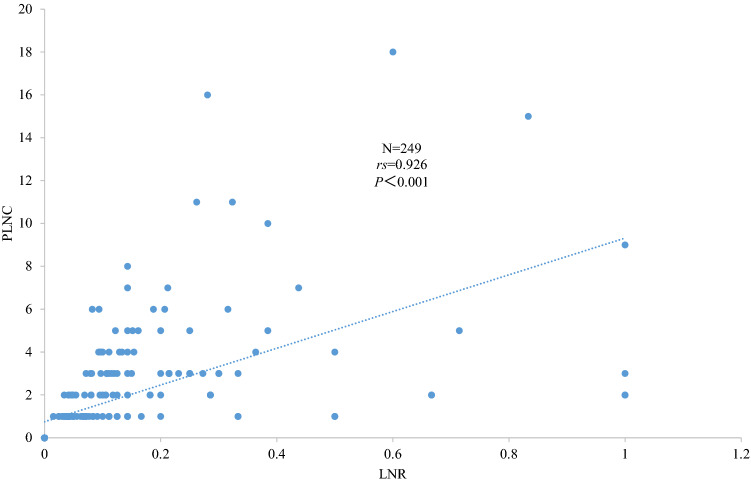
The correlation of LNR and PLNC in penile squamous cell carcinoma

Considering that the lymph nodes harvested from lymphadenectomy comprise positive and negative lymph nodes, PLNC is theoretically correlated with LNR. Thus, we analyzed the correlation between LNR and PLNC by performing a Spearman correlation analysis. The results (rs = 0.926, *P < *0.001) suggested that LNR and PLNC were significantly correlated (Fig. 3).

Next, univariate and multivariate Cox regression analyses were performed to investigate the independent prognostic factors influencing overall survival and cancer-specific survival. N stage, M stage, the 7th AJCC stage, lymph-vascular invasion, LNR, and PLNC (all *P < *0.1) were found to have a significant impact on both overall survival and cancer-specific survival while age and T stage (both *P < *0.1) only influenced overall survival (Table 3). Interestingly, no prognostic significance of pathological grade in PSCC was found, which was inconsistent with our general cognition of malignant tumors. A possible reason for this inconsistency is the limited sample size, hence the lack of adequate representation of the population.

**Table 3:** Univariate Cox regression analysis of overall survival and cancer-specific survival in PSCC

Variables	Overall survival			Cancer-specific survival		
	HR	95%CI	*P* value	HR	95%CI	*P* value
Age						
> 62 vs ≤ 62	1.548	(0.989, 2.422)	0.056*	1.134	(0.674, 1.908)	0.635
Marital status						
Single vs married	1.333	(0.772, 2.301)	0.302	1.367	(0.733, 2.549)	0.325
Unknown vs married	0.530	(0.128, 2.186)	0.380	0.38	(0.052, 2.777)	0.34
Divorced/separated/widowed vs married	1.314	(0.722, 2.394)	0.372	0.987	(0.457, 2.132)	0.973
T stage						
T2 vs T1a, T1b, T1NOS	1.243	(0.652, 2.371)	0.509	1.234	(0.593, 2.570)	0.574
T3 vs T1a, T1b, T1NOS	1.803	(0.953, 3.409)	0.070 *	1.492	(0.706, 3.152)	0.294
T4 vs T1a, T1b, T1NOS	0.000	(0.000, 2.993*10^215^)	0.964	0.000	(0.000, 1.018*10^267^)	0.970
N stage						
N1 vs N0	2.067	(1.028, 4.157)	0.042	3.599	(1.462, 8.858)	0.005
N2 vs N0	3.045	(1.531, 6.058)	0.002	5.051	(2.089, 12.215)	< 0.001
N3 vs N0	4.751	(2.712, 8.323)	< 0.001*	8.337	(3.912, 17.771)	< 0.001*
M stage						
M1 vs M0	8.254	(3.848, 17.705)	< 0.001*	9.749	(4.266, 22.280)	< 0.001*
The 7th AJCC stage						
II vs I	1.517	(0.203, 11.339)	0.685	0.670	(0.084, 5.355)	0.705
IIIA vs I	2.297	(0.294, 17.969)	0.428	1.722	(0.212, 13.997)	0.611
IIIB vs I	4.024	(0.526, 30.771)	0.180	3.122	(0.399, 24.403)	0.278
IV vs I	6.199	(0.850, 45.216)	0.072*	5.409	(0.738, 39.641)	0.097*
Histology						
Papillary squamous cell carcinoma vs Verrucous carcinoma	13130.765	(0.000, 4.438*10^52^)	0.868	18317.704	(0.000, 2.438*10^62^)	0.886
Squamous cell carcinoma, NOS vs Verrucous carcinoma	8664.636	(0.000, 2.880*10^52^)	0.874	8959.453	(0.000, 1.176*10^62^)	0.894
Squamous cell carcinoma, keratinizing, NOS vs Verrucous carcinoma	9033.025	(0.000, 3.003*10^52^)	0.873	9244.140	(0.000, 1.214*10^62^)	0.894
Squamous cell carcinoma, large cell, nonkeratinizing, NOS vs Verrucous carcinoma	13985.463	(0.000, 4.673*10^52^)	0.867	19733.170	(0.000, 2.601*10^62^)	0.885
Squamous cell carcinoma, spindle cell vs Verrucous carcinoma	10528.488	(0.000, 3.528*10^52^)	0.871	7593.573	(0.000, 1.011*10^62^)	0.896
Basaloid squamous cell carcinoma vs Verrucous carcinoma	3896.977	(0.000, 1.306*10^52^)	0.885	2927.710	(0.000, 3.896*10^61^)	0.907
Grade						
III–IV vs I–II	1.165	(0.726, 1.872)	0.527	1.201	(0.687, 2.100)	0.522
Primary site						
Glans penis vs prepuce	1.221	(0.477, 3.127)	0.678	1.691	(0.513, 5.579)	0.388
Body of penis vs prepuce	1.228	(0.371, 4.058)	0.737	1.544	(0.345, 6.905)	0.57
Overlapping lesion of penis vs prepuce	0.462	(0.090, 2.383)	0.356	0.774	(0.129, 4.635)	0.779
Penis, NOS vs prepuce	1.205	(0.470, 3.089)	0.698	1.322	(0.394, 4.436)	0.651
Lymph-vascular invasion						
Negative vs unknown	1.418	(0.630, 3.194)	0.399	1.570	(0.606, 4.068)	0.353
Positive vs unknown	3.028	(1.350, 6.792)	0.007*	2.697	(1.028, 7.078)	0.044*
LNR						
> 0.23 vs ≤ 0.23	4.291	(2.646, 6.959)	< 0.001*	5.351	(3.091, 9.262)	< 0.001*
PLNC						
> 1 vs ≤ 1				4.364	(2.538, 7.504)	< 0.001*
> 3 vs ≤ 3	3.914	(2.425, 6.318)	< 0.001*			
LNR(continuous)	22.315	(9.865, 50.474)	< 0.001*	28.274	(11.329, 70.565)	< 0.001*
PLNC(continuous)	1.165	(1.109, 1.224)	< 0.001*	1.187	(1.126, 1.251)	< 0.001*

*CI* confidence interval, *LNR* lymph node ratio, *PLNC* positive lymph node count

*Two-sided *P* value < 0.1

Multivariate Cox regression models for survival were used to compare the effects of LNR and PLNC. Considering that the 7th AJCC stage contains information on the N stage and M stage, several multivariate Cox regression models incorporating lymph-vascular invasion, the 7th AJCC stage, and LNR/PLNC were constructed (Table 4). We found that LNR (Model 1: HR = 2.788, 95%CI = (1.638, 4.745), *P < *0.001; Model 2: HR = 3.122, 95%CI = (1.725, 5.651), *P < *0.001) and lymph-vascular invasion (Positive vs Unknown: Model 1: HR = 3.023, 95% CI = (1.340, 6.817), *P = *0.008; Model 2: HR = 2.721, 95% CI = (1.031, 7.183), *P = *0.043) were independent prognostic factors for both overall survival and cancer-specific survival in both Model 1 and 2. Patients with LNR > 0.23 had a 3.122 fold higher probability of dying from PSCC than patients with LNR ≤ 0.23. Surprisingly, the 7th AJCC stage did not correlate with either overall survival or cancer-specific survival in both models. In Model 3, PLNC (HR = 2.298, 95% CI = (1.332, 3.963), *P = *0.003) and lymph-vascular invasion (Positive vs Unknown: HR = 2.731, 95% CI = (1.208, 6.177), *P = *0.016) were associated with overall survival, while the 7th AJCC stage was found to have no prognostic significance. However, all the three variables included in Model 4 exhibited no prognostic significance for cancer-specific survival. Therefore, LNR was found to be a more reliable prognostic factor for PSCC.

**Table 4:** Multivariate Cox regression analysis of overall survival and cancer-specific survival in PSCC

Variables	Overall survival			Cancer-specific survival		
	HR	95%CI	*P* value	HR	95%CI	*P* value
	Model 1			Model 2		
LNR						
> 0.23 vs ≤ 0.23	2.788	(1.638, 4.745)	< 0.001*	3.122	(1.725, 5.651)	< 0.001*
Lymph-vascular invasion						
Negative vs unknown	1.746	(0.764, 3.989)	0.186	2.094	(0.794, 5.524)	0.135
Positive vs unknown	3.023	(1.340, 6.817)	0.008 *	2.721	(1.031, 7.183)	0.043 *
The 7th AJCC stage						
II vs I	1.325	(0.175, 10.026)	0.785	0.663	(0.082, 5.362)	0.700
IIIA vs I	1.776	(0.222, 14.236)	0.589	1.518	(0.182, 12.694)	0.700
IIIB vs I	2.548	(0.326, 19.883)	0.372	2.100	(0.262, 16.829)	0.485
IV vs I	3.846	(0.513, 28.859)	0.190	3.780	(0.499, 28.634)	0.198
	Model 3					
PLNC						
> 3 vs ≤ 3	2.298	(1.332, 3.963)	0.003 *			
Lymph-vascular invasion						
Negative vs unknown	1.467	(0.643, 3.345)	0.362			
Positive vs unknown	2.731	(1.208, 6.177)	0.016 *			
The 7th AJCC stage						
II vs I	1.275	(0.168, 9.650)	0.814			
IIIA vs I	1.907	(0.238, 15.266)	0.543			
IIIB vs I	2.391	(0.303, 18.847)	0.408			
IV vs I	3.699	(0.490, 27.894)	0.204			
				Model 4		
PLNC						
> 1 vs ≤ 1				1.859	(0.837, 4.130)	0.128
Lymph-vascular invasion						
Negative vs unknown				1.719	(0.656, 4.501)	0.270
Positive vs unknown				2.419	(0.915, 6.396)	0.075
The 7th AJCC stage						
II vs I				0.636	(0.079, 5.147)	0.671
IIIA vs I				1.578	(0.189, 13.165)	0.673
IIIB vs I				1.637	(0.181, 14.832)	0.661
IV vs I				3.175	(0.386, 26.105)	0.283
	Model 5			Model 6		
LNR(continuous)	22.538	(7.818, 64.971)	< 0.001*	24.255	(7.194, 81.778)	< 0.001*
Lymph-vascular invasion						
Negative vs unknown	2.340	(0.988, 5.542)	0.053	2.943	(1.054, 8.214)	0.039
Positive vs unknown	4.044	(1.743, 9.379)	0.001*	3.861	(1.399, 10.652)	0.009*
The 7th AJCC stage						
II vs I	1.338	(0.177, 10.125)	0.778	0.671	(0.083, 5.431)	0.709
IIIA vs I	1.313	(0.163, 10.612)	0.798	1.140	(0.135, 9.610)	0.904
IIIB vs I	1.788	(0.227, 14.060)	0.581	1.473	(0.181, 11.962)	0.717
IV vs I	2.990	(0.396, 22.571)	0.288	3.062	(0.402, 23.324)	0.280
	Model 7			Model 8		
PLNC(continuous)	1.133	(1.055, 1.216)	0.001*	1.133	(1.050, 1.224)	0.001*
Lymph-vascular invasion						
Negative vs unknown	1.417	(0.620, 3.240)	0.409	1.563	(0.592, 4.123)	0.367
Positive vs unknown	2.824	(1.249, 6.384)	0.013*	2.442	(0.924, 6.458)	0.072
The 7th AJCC stage						
II vs I	1.230	(0.162, 9.324)	0.841	0.607	(0.075, 4.924)	0.640
IIIA vs I	1.604	(0.199, 12.913)	0.657	1.377	(0.164, 11.561)	0.768
IIIB vs I	1.823	(0.224, 14.834)	0.575	1.544	(0.183, 13.019)	0.690
IV vs I	3.200	(0.421, 24.318)	0.261	3.142	(0.408, 24.204)	0.272

*CI* confidence interval, *LNR* lymph node ratio, *PLNC* positive lymph node count

*Two-sided *P* value < 0.05

Although the superior prognostic value of LNR over PLNC was demonstrated, it was highly unlikely that patients with similar LNR/PLNC distributed on both sides of the cut-off value exhibited significantly different survival rate. The prognostic value of LNR and PLNC was further validated in univariate Cox regression analysis as continuous variables. Both LNR (continuous; For overall survival: HR = 22.315, 95%CI = (9.865, 50.474), *P < *0.001; For cancer-specific survival: HR = 28.274, 95%CI = (11.329, 70.565), *P < *0.001) and PLNC (continuous; For overall survival: HR = 1.165, 95% CI = (1.109, 1.224), *P < *0.001; For cancer-specific survival: HR = 1.187, 95% CI = (1.126, 1.251), *P < *0.001) exhibited influence on survival (Table 3). In multivariate Cox regression analysis, LNR (continuous; For overall survival: HR = 22.538, 95% CI = (7.818, 64.971), *P < *0.001; For cancer-specific survival: HR = 24.255, 95% CI = (7.194, 81.778), *P < *0.001) and PLNC (continuous; For overall survival: HR = 1.133, 95% CI = (1.055, 1.216), *P = *0.001; For cancer-specific survival: HR = 1.133, 95% CI = (1.050, 1.224), *P = *0.001) were found to be significantly associated with overall survival and cancer-specific survival (Table 4).

### Subgroup and survival analysis in node-positive patients

Subgroup analysis was carried out to assess the association between clinical factors and survival in 132 patients with positive lymph nodes (Tables 5 and 6). Univariate analysis revealed that the N stage, M stage, the 7th AJCC stage, lymph-vascular invasion, LNR, and PLNC (all *P < *0.1) were associated with both overall survival and cancer-specific survival whereas age and T stage (both *P < *0.1) were only associated with overall survival. Multivariate analysis demonstrated that, LNR was an independent prognostic factor for both overall survival and cancer-specific survival (Model 1: HR = 2.612, 95% CI = (1.529, 4.461), *P < *0.001; Model 2: HR = 2.994, 95% CI = (1.647, 5.440), *P < *0.001) while PLNC (Model 4: HR = 1.447, 95% CI = (0.645, 3.248), *P = *0.370) was not significantly associated with cancer-specific survival. These results suggested that LNR exhibited better prognostic value compared with PLNC in node-positive patients.

**Table 5:** Univariate Cox regression analysis of overall survival and cancer-specific survival in patients with positive lymph nodes

Variables	Overall survival			Cancer-specific survival		
	HR	95%CI	*P*	HR	95%CI	*P*
Age						
> 62 vs ≤ 62	1.652	(0.984, 2.774)	0.058*	1.223	(0.694, 2.155)	0.487
Marital status						
Single vs married	1.254	(0.673, 2.336)	0.476	1.258	(0.642, 2.468)	0.504
Unknown vs married	0.331	(0.045, 2.428)	0.277	0.437	(0.059, 3.207)	0.415
Divorced/separated/widowed vs married	0.996	(0.493, 2.012)	0.990	0.717	(0.298, 1.727)	0.458
T stage						
T2 vs T1a, T1b, T1NOS	1.364	(0.610, 3.051)	0.450	1.422	(0.605, 3.347)	0.420
T3 vs T1a, T1b, T1NOS	2.257	(1.030, 4.945)	0.042*	1.810	(0.765, 4.287)	0.177
T4 vs T1a, T1b, T1NOS	< 0.001	(0.000, 2.707*10^256^)	0.972	0.000	(0.000, 7.307*10^277^)	0.974
N stage						
N2 vs N1	1.361	(0.637, 2.907)	0.427	1.336	(0.566, 3.149)	0.509
N3 vs N1	2.103	(1.104, 4.004)	0.024*	2.193	(1.060, 4.539)	0.034*
M stage						
M1 vs M0	4.925	(2.268, 10.695)	< 0.001*	5.274	(2.292, 12.134)	< 0.001*
The 7th AJCC stage						
IIIB vs IIIA	1.641	(0.716, 3.758)	0.242	1.728	(0.657, 4.548)	0.268
IV vs IIIA	2.853	(1.414, 5.755)	0.003*	3.338	(1.466, 7.597)	0.004*
Histology						
Squamous cell carcinoma, NOS vs Papillary squamous cell carcinoma	0.330	(0.045, 2.435)	0.277	0.280	(0.038, 2.084)	0.214
Squamous cell carcinoma, keratinizing, NOS vs Papillary squamous cell carcinoma	0.415	(0.055, 3.123)	0.393	0.333	(0.044, 2.551)	0.290
Squamous cell carcinoma, large cell, nonkeratinizing, NOS vs Papillary squamous cell carcinoma	0.490	(0.051, 4.746)	0.538	0.513	(0.053, 4.980)	0.565
Squamous cell carcinoma, spindle cell vs Papillary squamous cell carcinoma	0.237	(0.015, 3.843)	0.311	0.257	(0.016, 4.163)	0.339
Basaloid squamous cell carcinoma vs Papillary squamous cell carcinoma	0.203	(0.013, 3.275)	0.261	0.213	(0.013, 3.436)	0.276
Grade						
Grade III, Grade IV vs Grade I, Grade II	1.023	(0.601, 1.741)	0.934	1.062	(0.587, 1.920)	0.842
Primary site						
Glans penis vs prepuce	2.164	(0.656, 7.139)	0.205	2.791	(0.658, 11.850)	0.164
Body of penis vs prepuce	1.364	(0.274, 6.777)	0.704	2.152	(0.359, 12.885)	0.401
Overlapping lesion of penis vs prepuce	0.926	(0.155, 5.543)	0.933	1.409	(0.198, 10.006)	0.732
Penis, NOS vs prepuce	1.945	(0.586, 6.464)	0.277	2.208	(0.512, 9.517)	0.288
Lymph-vascular invasion						
Negative vs unknown	1.499	(0.615, 3.656)	0.373	2.126	(0.738, 6.129)	0.163
Positive vs unknown	2.665	(1.105, 6.425)	0.029*	2.771	(0.946, 8.111)	0.063*
LNR						
> 0.23 vs ≤ 0.23	2.769	(1.647, 4.656)	< 0.001*	2.988	(1.678, 5.321)	< 0.001*
PLNC						
> 1 vs ≤ 1				1.980	(1.027, 3.818)	0.041*
> 3 vs ≤ 3	2.476	(1.472, 4.163)	0.001*			

*CI* confidence interval, *LNR* lymph node ratio, *PLNC* positive lymph node count

*Two-sided *P* value < 0.1

**Table 6:** Multivariate Cox regression analysis of overall survival and cancer-specific survival in patients with positive lymph nodes

Variables	Overall survival			Cancer-specific survival		
	HR	95%CI	*P*	HR	95%CI	*P*
	Model 1			Model 2		
LNR						
> 0.23 vs ≤ 0.23	2.612	(1.529, 4.461)	< 0.001*	2.994	(1.647, 5.440)	< 0.001*
Lymph-vascular invasion						
Negative vs unknown	1.598	(0.641, 3.983)	0.315	2.333	(0.790, 6.890)	0.125
Positive vs unknown	2.440	(0.994, 5.986)	0.051	2.545	(0.856, 7.570)	0.093
The 7th AJCC stage						
IIIB vs IIIA	1.319	(0.567, 3.069)	0.520	1.281	(0.475, 3.449)	0.625
IV vs IIIA	2.178	(1.060, 4.477)	0.034*	2.513	(1.083, 5.835)	0.032*
	Model 3					
PLNC						
> 3 vs ≤ 3	2.092	(1.2309, 3.620)	0.008*			
Lymph-vascular invasion						
Negative vs Unknown	1.232	(0.497, 3.053)	0.653			
Positive vs Unknown	2.067	(0.841, 5.081)	0.113			
The 7th AJCC stage						
IIIB vs IIIA	1.189	(0.496, 2.854)	0.698			
IV vs IIIA	2.000	(0.945, 4.233)	0.070			
				Model 4		
PLNC						
> 1 vs ≤ 1				1.447	(0.645, 3.248)	0.370
Lymph-vascular invasion						
Negative vs unknown				1.831	(0.628, 5.342)	0.268
Positive vs unknown				2.207	(0.745, 6.543)	0.153
The 7th AJCC stage						
IIIB vs IIIA				1.182	(0.365, 3.832)	0.780
IV vs IIIA				2.373	(0.885, 6.366)	0.086

*CI* confidence interval, *LNR* lymph node ratio, *PLNC* positive lymph node count

*Two-sided *P* value < 0.05

However, the absence of the 7th AJCC stage in all multivariate Cox proportional hazards regression models indicated that there were defects in our models. Large-scale analysis of complete and representative patients’ information is needed for the calibration of the Cox regression model. In conclusion, LNR exhibited a better prognostic prediction than PLNC and could thus, serve as a promising prognostic factor in PSCC.

## Discussion

Penile cancer is a relatively rare disease worldwide, it accounts for only 1% of all male malignancies, but causes considerable psychological and physiological trauma [1]. Squamous cell carcinoma is the most common histological type of penile cancer, with approximately 80% of cases localized in the glans penis and prepuce [13]. The diagnosis, treatment, and prognosis of PSCC are greatly correlated with lymph node status. The diagnosis of PSCC is mainly based on physical examination and regional lymph nodes evaluation [14]. Surgical resection with regional lymph node dissection remains the standard therapeutic modality for locally advanced cases [15] and an important prognostic values [16, 17]. However, apart from metastasis, lymphadenopathy may also be caused by infection [18]. Thus, antibiotic treatment can prevent unnecessary lymph node biopsy. Besides, the lymphatic nodal metastasis status is the most significant prognostic factor in patients with penile squamous cell carcinoma [19]. Although men with less severe disease exhibit prolonged survival, the prognosis of advanced or metastatic PSCC remains poor, thus requiring a more robust prognostic index than the traditional AJCC TNM staging system.

LNR and PLNC have exhibited prognostic value in a variety of tumors, including salivary gland cancer [20], prostate cancer [21], non-small cell lung cancer [22, 23], breast cancer [24], and colon cancer [25]. To date, the association between LNR/PLNC and survival in patients with PSCC has not been well elucidated. Svatek et al. conducted a survey with 45 patients between 1979 and 2007 and reported that LNR (≤ 6.7 vs. > 6.7%) was significantly associated with CSS in patients with node-positive PSCC [26]. However, the small population and excessively long period limited the validity of the findings. Similarly, Lughezzani et al. observed significant differences in survival rates based on LNR [27]. The highlighted studies stratified survival outcomes using the median value, which is not very rigorous in determining the threshold. Besides, both studies did not compare the prognostic value of LNR and PLNC.

A recent study proposed that LNR was a better prognostic indicator compared to PLNC [28], however, the study population comprised only 28 penile cancer patients. Another study suggested that LNR, but not PLNC was a predictor of dismal survival outcomes in node-positive PSCC [29]. However, the study simultaneously added LNR and PLNC into the multivariate Cox regression model, which led to multicollinearity.

Compared with previous studies, the present study had a relatively large sample size, thus making it more applicable in clinical practice. Furthermore, there is still a considerable discrepancy in the threshold of LNR to discriminate between favorable and poor survival in prior studies, ranging between 0.067 and 0.33 [26–30]. This discrepancy may be explained by the use of varied surgical approaches, the extent of lymph node dissection, and the use of different statistical methods to calculate the optimal cut-off values. Unlike in previous studies where stratification of patients was done using the median, the optimal cut-off values for LNR/PLNC were determined using the X-tile program in this study. The optimal cut-off points were 0.23 for LNR (for both OS and CSS) and 3 (for OS)/1 (for CSS) for PLNC. When considered as categorical variables in both univariate and multivariate Cox regression analyses, LNR > 0.23 predicted worse survival outcomes compared with LNR ≤ 0.23 in patients with penile cancer. However, PLNC did not exhibit prognostic value in multivariate model predicting CSS. Thus, LNR was found to be a better prognostic factor for PSCC. Surprisingly, when converted to continuous variables, both LNR and PLNC were found to be independent prognostic factors of poor survival in penile cancer. The varying prognostic value exhibited by PLNC may be attributed to the loss of information contained in the raw data when converted to a categorical variable. However, a definite threshold is required by clinicians assessing prognosis in clinical practice. Therefore, investigating the prognostic value of LNR and PLNC as categorical variables makes it easier for clinical decision-making. Considering that heterogeneity may exist in patients with and without lymph node metastasis, subgroup analysis was performed in node-positive patients. LNR (*P < *0.001) was found to be a better prognostic marker than PLNC (*P = *0.370) for CSS. Accordingly, LNR was found to be a better predictor for survival than PLNC in patients with PSCC, and could also be used to distinguish between postoperative PSCC patients with poor prognosis requiring adjuvant therapy. The optimal cut-off value of 0.23 for LNR may not be directly used in clinical practice, and further consideration in combination with clinical information is needed.

The prognostic value of PLNC depends to a great extent on surgical and pathological procedures. In conditions of inadequate lymph node dissection, this can lead to the phenomenon of “stage migration” [31]. The superior prognostic value of LNR can be explained by the incorporation of disease burden and quality pathologic examination (PLNC), and the extent of lymphadenectomy (the number of examined nodes), which would reduce bias due to insufficient lymph node evaluation [32, 33]. Thus, in the case of sufficient lymph node retrieval, the prognostic value of LNR may decline and PLNC would more precisely reflect the nodal status. In the present study, LNR was found to be a better predictor for long-term survival compared with PLNC in PSCC, thus, reflecting insufficient clinical lymph node dissection. Therefore, standardization of lymphadenectomy is needed in clinical practice.

Despite the advantages of the present study, there were several potential limitations. First, this was a retrospective study based on a public database, and the study population was highly selected, which may result in selection bias. Second, the data obtained from the SEER database involved multiple centers. Thus, standardization of the surgical approach, especially the extent of lymphadenectomy, could not be implemented due to the multicentre nature of the study. Third, several important variables were not included in the SEER database, such as the size of lymph nodes, the region of lymph node metastasis (inguinal/pelvic lymph nodes), extranodal extension, tumor recurrence, and adjuvant therapy. These confounding factors influence survival but could not adjusted in our study. Finally, some common prognostic factors such as T stage and pathological grade were not associated with CSS in this study, possibly because the sample size of some stratified patients was too small.

## Conclusion

In summary, we demonstrated that LNR is associated with the long-term survival of postoperative PSCC patients and is a better prognostic marker than PLNC. Besides, LNR can be used to stratify patients for adjuvant therapy in the case of inadequate lymph node dissection.

## Data Availability

The datasets generated during and/or analysed during the current study are available from the corresponding author on reasonable request.
